# Postoperative Neutrophil to Lymphocyte Ratio as an Overall Mortality Midterm Prognostic Factor following OPCAB Procedures

**DOI:** 10.3390/clinpract11030074

**Published:** 2021-09-03

**Authors:** Tomasz Urbanowicz, Michał Michalak, Aleksandra Gąsecka, Bartłomiej Perek, Michał Rodzki, Michał Bociański, Ewa Straburzyńska-Migaj, Marek Jemielity

**Affiliations:** 1 Department of Cardiac Surgery and Transplantology, Poznan University of Medical Sciences, 61-848 Poznan, Poland; bperek@ump.edu.pl (B.P.); michal.rodzki@skpp.edu.pl (M.R.); michal.bocianski@skpp.edu.pl (M.B.); mjemielity@poczta.onet.pl (M.J.); 2 Department of Computer Science and Statistics, Poznan University of Medical Sciences, 60-806 Poznan, Poland; michal@ump.edu.pl; 3 Department of Cardiology Medical University of Warsaw, 02-091 Warsaw, Poland; gaseckaa@gmail.com; 4 Ist Department of Cardiology, Poznan University of Medical Sciences, 61-848 Poznan, Poland; ewa.straburzynska-migaj@skpp.edu.pl

**Keywords:** NLR 1, OPCAB 2, platelets 3, LVEF 4

## Abstract

Background: Off-pump coronary artery bypass grafting (OPCAB) is believed to limit inflammatory reaction. Neutrophil to lymphocyte ratio (NLR) is one of the more common and easily accessible markers of inflammatory response. The aim of the study was to compare postoperative results of NLR with mid-term OPCAB results. Methods: In total, 224 patients (198 (88%) men and 26 (12%) women) with mean age 65 +/− 9 years who underwent OPCAB though median full sternotomy in our department in 2018 enrolled into the study. We scrupulously collected the postoperative mid-term results, including survival rate, clinical status and risk for major adverse events, and compared them with perioperative laboratory results. Results: A three-year follow-up was completed by 198 individuals (90% survival rate) with 12 (5%) showing major adverse cardiovascular (MACE) events risk. In the multivariable analysis, the laboratory parameters noticed on the 1st postoperative day were statistically significantly predictive of survival, including neutrophils (HR 1.59, 1.33–1.89 95%CI, *p* < 0.0001), platelets (HR 1.01, 1.01–1.01 95%CI, *p* = 0.0065), NLR (HR 1.47, 1.3–1.65 95%CI, *p* < 0.0001) and postoperative ejection fraction (HR 0.9, 0.87–0.95 95%CI, *p* < 0.0001). Conclusions: Postoperative NLR above 4.6, as an inflammatory reaction marker, is related to mid-term mortality in OPCAB patients.

## 1. Introduction

The surgical revascularization of coronary artery disease can be performed with cardiopulomnary bypass, or with the “beating heart” technique called off-pump coronary artery bypass grafting (OPCAB). It allows one to omit the cardiopulmonary circuit, along with all possible side effects, including inflammatory reaction activation [1].

Off-pump surgical revascularization of complex coronary artery disease has gained worldwide attention [2,3,4]. The major advantage of off-pump coronary artery bypass grafting (OPCAB) consists in the avoidance of non-physiological blood flow and minimization of the risk of complications related to inflammatory response. Inflammation plays a significant role in atherosclerosis progression and its complications [5,6].

One of the more easily accessible markers of inflammatory response is neutrophil-to-lymphocyte ratio (NLR). Its capacity to predict adverse outcomes in patients with coronary artery disease has already been postulated [7].

The aim of the study was to compare postoperative results of NLR with mid-term OPCAB results, since there are no medical references evaluating the correlation between postoperative NLR and mid-term mortality risk in off-pump surgery.

## 2. Materials and Methods

### 2.1. Study Population

In total, 224 patients (198 (88%) men and 26 (12%) women) with a mean age of 65 ± 9 years who had undergone OPCAB (off-pump coronary artery bypass grafting) through median full sternotomy at our Department in 2018 were enrolled into the study. Ethical approval was obtained from the local university’s bioethics committee (273/21). All patients were provided with a form of written informed consent for a surgical procedure, and the research was conducted according to the principles laid down in the Declaration of Helsinki.

The presented group was subdivided in terms of 3-year survival into 2 subgroups. Detailed demographical and clinical information are presented in Table 1.

The mean follow-up time was 2.5 years. The data were collected from initial hospitalization, followed by regular checks at an outpatient clinic and telephone survey. The exclusion criteria included temporary mechanical support prior to surgery, including intraortic balloon counterpulsation, the need for respiratory support, kidney failure requiring hemodialysis, a previous cardiac surgery, an active infection, or an acute phase of myocardial infarction. Chronic inflammatory diseases and a history of oncological diseases were also added to the exclusion criteria.

### 2.2. Patient and Public Partnership

The patients were first enlisted into the study by verification of their survival, and their participation was voluntary, subject to being informed of the study. Survival was additionally assessed using national electronic verification data available for our country.

### 2.3. Laboratory Analysis

Laboratory results were obtained prior to surgery, as well as on day 1 and day 7 following the surgery. The perioperative laboratory results are presented in Table 2. The whole blood count was the only standard laboratory test presenting inflammatory reaction on the 1st and 7th postoperative days. The other inflammatory parameters, such as C-reactive protein (CRP) or procalcitonine, were assessed only if infection was suspected.

Postoperative mid-term results, including survival rate, clinical status, and the risk of major adverse events, were meticulously collected.

Postoperative myocardial infarction (type 5) diagnosis was based on the following criteria: elevated serum Troponin-I levels combined with a decrease in left ventricle ejection fraction in accordance with fourth universal definition [8].

### 2.4. Surgical Technique

Procedures were performed through median sternotomy without cardiopulmonary bypass application (OPCAB technique) by a team of experienced surgeons. Prior to performing anastomosis, heparin was administered according to the results of ACT (activating clotting time) with a mean value of 458 ± 42 s. The Octopus III (Medtronic, Minneapolis, MN, USA) local stabilizer was used in combination with a deep pericardial stitch to elevate the beating heart. Following the application of intraluminal shunts into coronary arteries, the anastomoses were performed with a monofilament 7-0 suture. A double dose of protamine was routinely administered after performing the anastomoses in order to reverse heparin action (mean ACT 132 ± 36 s).

### 2.5. Clinical Endpoint

To identify the effect of the post-procedural NLR on mid-term mortality in patients undergoing off-pump surgical revascularization.

### 2.6. Statistical Analysis

Continuous variables were reported as mean ± standard deviation (SD). Categorical data were presented as numbers and percentages. The comparison of interval parameters between the survivors and the deceased group was performed by Student’s t-test, and, if the data did not follow the normal distribution, the Mann–Whitney test was used as an alternative. Categorical data were analyzed using the test of proportions. The Cox’s proportional hazard regression model was used to check whether the analyzed demographical and clinical data could be a risk factor for all-cause mortality. Both univariate and multivariate analyses were performed. Additionally, for significant factors, the ROC analysis was performed to find the optimal cut-off point for continuous parameters. In the next step, a multivariable Cox’s model was used, wherein all data were binary. The analysis was performed using MedCalc^®^ Statistical Software version 19.6 (MedCalc Software Ltd., Ostend, Belgium). All tests were considered significant at *p* < 0.05.

## 3. Results

There were two (1%) perioperative deaths and seven (3%) myocardial infarctions in the presented groups. A three-year follow-up was not completed by 24 individuals, with a 10% mortality rate, and major adverse cardiovascular (MACE) events combined with percutaneous interventions were found in 12 (5%) more patients.

There were 26 deaths in the presented analysis related to 15 (58%) coronary syndromes and 11 (42%) secondary to stroke.

The two groups (the deceased (non-survivors) and the survivors) were statistically different regarding concomitant diseases, including: history of stroke (*p* < 0.0001), peripheral artery disease (*p* = 0.0008), and left ventricle ejection fraction (*p* = 0.002).

The median values of the maximum Troponin-I serum level following the surgery were 4 ± 6 vs. 9.9 ± 11 mcg/L in groups 1 and 2, respectively. Detailed results are presented in Table 2.

The preoperative NLR results were comparable in both groups (3.3 ± 1.8 vs. 3.2 ± 1.5 (*p* = 0.7119) in the survivors vs. the deceased group, respectively). A postoperative NLR increase on post-op day 1 was observed in the survivors and the deceased group (2.8 ± 1.6 vs. 5.1 ± 3.6 (*p* = 0.0003) and 2.7 ± 1.4 vs. 3 ± 1.9 (*p* = 0.6694), respectively). The NLR increase on post-op day 1 decreased back to preoperative values on day 7. The preoperative NLR and its values on post-op day 7 in the survivors group was lower (3.3 ± +/−1.8 vs. 2.7 ± 1.4 (*p* = 0.0002) and comparable in the deceased group, 3.2 ± 1.5 vs. 3 ± 1.9 (*p* = 0.6754).

Interestingly, the maximal serum levels of myocardial injury markers (Troponin-I) were comparable between both groups (4 ± 6.3 mcg/L and 9.9 ± 11 mcg/L (*p* = 0.1206)). Preoperative and postoperative whole blood count data, including the commonly accepted marker of inflammatory reaction, such as neutrophil-to-lymphocyte ratio (NLR), are presented in Table 2.

Univariable and multivariable Cox’s proportional hazard regression analysis was performed and is presented in Table 3. 

In the univariable analysis, stroke in medical history (HR 14.07, 6.34–31.22, 95%CI, *p* < 0.0001) and peripheral artery disease (HR 3.9, 1.77–8.6 95% CI, *p* = 0.0007) were found to be significant demographical factors affecting survival. Postoperative echocardiographic results, including left ventricle diastolic dimension (HR 1.08, 1.02–1.16, 95%CI, *p* = 0.0191) and ejection fraction (HR 0.88, 0.85–0.91, 95%CI, *p* < 0.0001). Interestingly, among the laboratory parameters, those measured on post-op day 1 were statistically significantly predictive of survival, including neutrophils (HR 1.59, 1.33–1.89 95%CI, *p* < 0.0001), platelets (HR 1.01, 1.01–1.01 95%CI, *p* = 0.0065), and NLR (HR 1.47, 1.3–1.65 95%CI, *p* < 0.0001).

**Figure 1:**
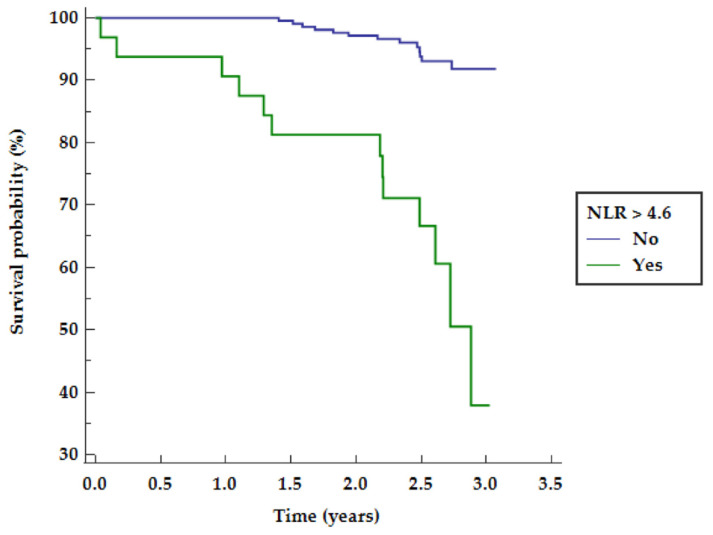
NLR probability of mid-term survival. NLR—neutrophil to lymphocyte ratio.

NLR above 4.6 was found to be related to mid-term survival as presented in Figure 1.

Postoperative ejection fraction (HR 0.9, 0.87–0.95 95%CI, *p* < 0.0001) and laboratory parameters noted on post-op day 1 were found to represent significant factors in the multivariable analysis (Table 3).

**Figure 2:**
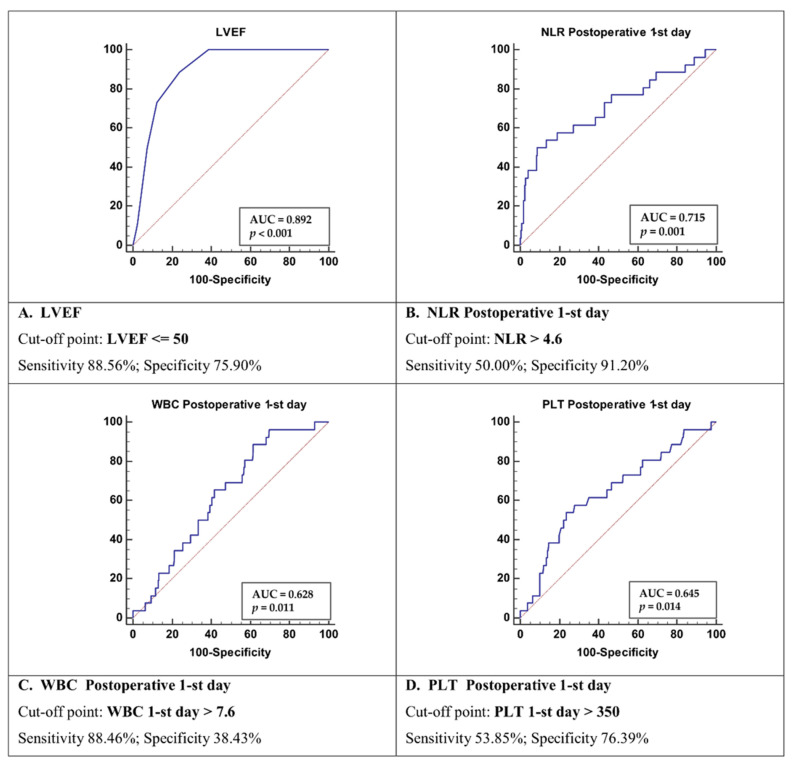
Receiver operating characteristic curves for Cox’s model LVEF (**A**). NLR on first postoperative day (**B**). WBC on first postoperative day (**C**). PLT on first postoperative day (**D**). Abbreviations: LVEF—left ventricle ejection fraction, NLR—neutrophil to lymphocyte ratio, PLT—platelets count, WBC—white blood count.

Receiver operating characteristic curves for Cox’s model revealed the following significant parameters: LVEF ≤ 50% with AUC = 0.892 (sensitivity 88.6% and specificity 76%), WBC above 7.6 10E9/L with AUC = 0.628 (sensitivity 88.5% and specificity 38%), Plt above 350 k/L with AUC = 0.645 (sensitivity 54% and specificity 76%), and NLR above 4.6 with AUC = 0.715 (sensitivity 50% and specificity 91%) (Figure 2).

The multivariable Cox’s proportional hazard regression analysis was performed once again, taking all significant parameters as a binary. The results were presented in Table 4, including left ventricle ejection fraction ≤50% (HR 12.6, 3.69–42.72 95%CI, *p* = 0.0001), WBC > 7.6 (HR 1.04, 0.32–3.45 95%CI, *p* = 0.9409), platelets > 350 k/L (HR 2.7, 1.19–6.15 95%CI, *p* = 0.018) and NLR > 4.6 (HR 9.3, 3.6–24.02 95%CI, *p* < 0.0001).

## 4. Discussion

The unique result of the study is the relationship between the NLR increase on the first postoperative days after off-pump surgical revascularization and mid-term survival in multivariable analysis. In our analysis, four parameters were found to be related to mid-term mortality, including postoperative ejection fraction below 50%, NLR above 4.6, WBC above 7.6, and platelet count above 350 k per liter. The strongest predictor for mid-term survival was left ventricle ejection fraction (AUC = 0.895). The results of our study present the NLR as an independent mortality predictor following the OPCAB procedure with moderate significance (AUC = 0.715). This study points out the significance of inflammatory reaction activation as one of the possible prognostic factors.

Surgical stress by cellular immunity suppression may result in postoperative systemic leukocytic alterations, including leukocytosis, neutrophilia, lymphopenias, or inflammatory mediator overproduction [9].

The high NLR (above 4.6) on post-op day 1 was positively correlated with 3-year mortality. The results indicate that inflammatory processes occurring one day after the surgery have a significant negative impact on the patient’s survival. The postoperative NLR results were related to ICU stay and length of hospitalization in previous studies [10]. The correlation between the risk of MACCE following surgical revascularization in CPB was presented with a cut-off value of 4.32 in the multivariable analysis [11]. We present the results of the study with a similar cut-off value for mid-term mortality as the end-point analysis. We would like to point out that NLR reverted to preoperative values on post-op day 7. This suggests that after a surgical procedure, inflammatory processes are temporarily activated, and may trigger an unknown cascade leading to increased mortality within 2.5 years’ time. The overactivation of inflammatory system was transient, but further investigation seems to be required to distinguish between the prevalence of the patients’ hyperreactivity and a single inducible reaction. The association between increased preoperative NLR and cardiovascular risk, as well as cardiovascular and cerebrovascular events (MACE), in the 30-day postoperative period in non-cardiac surgery was presented by Larman [12].

Tan in his review postulated the association of increased preoperative values of NLR (>3.3 in cardiac surgery) with increased mortality at a mean follow-up of 34.8 months [13].

Increased NLR represents neutrophil activation combined with lymphocyte depletion. Neutrophils, as short-lived phagocytic cells, are characterized by a broad spectrum of biologically active molecules (myeloperoxidase, proteinases) [14]. According to Zernecke, the increased neutrophil content in plaques is associated with apoptosis and a proinflammatory phenotype [15]. In his study, the activation of circulating neutrophils was investigated, as labeled neutrophils were noted in atherosclerotic plaques after 8 weeks.

Myocardial ischemia/reperfusion injury occurring in the early postoperative phase involves the activation of neutrophils (and other cellular blood elements) combined with complementary system activation and molecular oxygen [16]. Lymphopenia, as observed in high NLR values, is another independent factor for atherosclerosis progression, as presented in previous studies [17,18].

The results of our study found a statistically significant relationship between WBC and survival in multivariable analysis. There are other studies in the literature presenting total white blood cell count (WBC) as a mortality predictor after surgical revascularization [19]. Other variables in outcome prediction include subtypes of WBC or specific ratios, such as the neutrophil-to-lymphocyte ratio [20]. If the whole blood count parameters are easily affected by the patient’s hydration level, NLR is believed to be relatively stable.

Contrarily, the Troponin-I serum level, a commonly used marker for myocardial injury, was not found in our results to be a mid-term mortality risk factor. This corroborates with the results obtained by Yan Li [21].

We present the relationship between perioperative NLR ratio and 3-year survival as the perioperative indicator of inflammatory response to the procedure. Chronic inflammatory processes are linked to atherosclerosis progression and plaque rupture, as presented in the review by Yuhua Zhu [22]. Inflammation initiates and promotes the development of atherosclerotic changes. We believe that the increased inflammatory activation during the perioperative period may strongly affect results, since surgical injury induces endogenous mediators that alter the immuno-inflammatory response [23].

Avoiding cardiopulmonary bypass during surgical revascularization has been proven to limit inflammatory response [24,25]. Contrarily, the complementary cascade of activation was on a similar level, according to Ascione’s study [26]. Additionally, neurohumoral activation triggering the systemic stress response was presented in a prospective study by Velissaris [27]. Shulze revealed the significantly lower expression of the TNF-system and Il-2r in OPCAB patients compared to CBP, except for similar Il-6 levels [28].

Postoperative high serum levels of C-reactive protein—another inflammatory marker commonly used in clinical practice—were found by Min [29] to be associated with a risk of MACE in the long-term.

Our study results present a negative correlation between left ventricle ejection fraction and mid-term survival. Decreased LVEF levels below 50% were found to be a mortality predictor. Postoperative deterioration of the left ventricle ejection fraction represents a myocardial perioperative injury significantly related to long-term survival, as postulated in previous studies [30,31].

The results from our retrospective analysis present the link between inflammatory reaction activation and risk for mid-term mortality. The NLR was found be a marker of moderate significance in the presented study. The significance of NLR in the perioperative period of cardiovascular procedures was already presented [32]. Nevertheless, the deaths were related to cardiovascular complications with known linkages to inflammatory processes [22].

We present a statistically significant relationship between the platelet count and mortality risk. Increased platelet counts are closely related to inflammatory reactions and increased NLR [33]. Contact between platelets and neutrophils modulates the thrombotic and inflammatory reaction [34]. High NLR may also increase platelet activity in the patient after coronary artery interventions, and both combined may help identify the patients with high risk of recurrent acute syndrome [35,36]. Therefore, we believe that the close relationship between platelets and NLR indicate that one of them should be considered a predictor.

This was a retrospective study, but it highlights possible new approaches to patients undergoing OPCAB procedures. We intend to follow up more closely this group of patients (NLR > 4.6) with more frequent tests, including treadmill tests and angiographies, if indicated. This group will be more closely monitored regarding cardiovascular risk factors, including hypertension.

## 5. Conclusions

Postoperative increases in NLR, as an inflammatory reaction marker, are related to mid-term mortality in OPCAB patients.

## Figures and Tables

**Table 1:** Demographical and clinical data.

Parameter	Group 1 Survivors (*n* = 198)	Group 2 Deceased (*n* = 26)	*p*-Value
Gender M/F	174 (88%)/25 (12%)	24 (92%)/2 (8%)	*p* = 0.5483
Age	65 +/− 9	67 +/− 9	*p* = 0.2869
Concomitant diseases:			
1. arterial hypertension	166 (84%)	21 (81%)	*p* = 0.6973
2. DM	78 (39%)	9 (65%)	*p* = 0.0116
3. Stroke	5 (3%)	8 (31%)	***p* < 0.0001**
4. Hypercholesterolemia	134 (68%)	18 (70%)	*p* = 0.8368
5. PAD	29 (15%)	11 (42%)	***p* = 0.0008**
Surgical indication:			*p* = 0.5650
1. LM disease	102 (52%)	12 (46%)	*p* = 0.5629
2. 3 vessels disease	88 (44%)	13 (50%)	*p* = 1.0000
3. 2 vessels disease	8 (4%)	1 (4%)	
Echocardiographic results			
1. LV diameter (mm)	47 +/− 6	48 +/− 6	*p* = 0.4257
2. LVEF (%)	54 +/− 8	50 +/− 7	***p* = 0.0159**
Surgery:			
1. overall time (min)	141 +/− 42	139 +/− 39	*p* = 0.8182
2. Mean anastomosis	2.3 +/− 0.7	2.3 +/− 0.7	*p* = 1.0000
Hospitalization time (days)	8.8 +/− 3	13 +/− 10	***p* = 0.0433**
(excluding in hospital mortality)		(10 +/− 4)	

Abbreviations: COPD—chronic obstructive pulmonary disease, DM—diabetes mellitus, LV—left ventricle, LVEF—left ventricle ejection fraction, PAD—peripheral artery disease.

**Table 2:** Perioperative laboratory results.

Parameters	Group 1 Survivors (*n* = 198)	Group 2 Deceased (*n* = 26)	*p*-Value
Preoperative:			
1. WBC, ×10^9^/L (mean ± SD)	8.4 +/− 3.3	7.7 +/− 1.9	*p* = 0.6780
2. Neutrophils, ×10^9^/L (mean ± SD)	5.3 +/− 1.7	5.1 +/− 1.5	*p* = 0.7849
3. Lymphocyte, ×10^9^/L (mean ± SD)	2.2 +/− 2.5	1.8 +/− 0.7	*p* = 0.4513
4. Hb, mmol/L (mean ± SD)	8.7 +/− 0.9	8.6 +/− 1.1	*p* = 0.9893
5. Plt, ×10^9^/L (mean ± SD)	229 +/− 63	233 +/− 63	*p* = 0.9397
6. NLR (mean ± SD)	3.3 +/− 1.8	3.2 +/− 1.5	*p* = 0.7119
7. Troponin, ng/mL (mean ± SD)	0.23 +/− 2.8	0.02 +/− 0.4	*p* = 0.1442
Postoperative 1st day):			
1. WBC, ×10^9^/L (mean ± SD)	9.1 +/− 5	12.1 +/− 13	***p* = 0.0331**
2. Neutrophils, ×10^9^/L (mean ± SD)	5.2 +/− 2	8.7 +/− 11	***p* = 0.0012**
3. Lymphocyte, ×10^9^/L (mean ± SD)	2.5 +/− 3.4	1.8 +/− 0.7	*p* = 0.0779
4. Hb, mmol/L (mean ± SD)	6.9 +/− 0.6	7 +/− 0.5	*p* = 0.3570
5. Plt, ×10^9^/L (mean ± SD)	304 +/− 92	354 +/− 107	***p* = 0.0157**
6. NLR (mean ± SD)	2.8 +/− 1.6	5.1 +/− 3.6	***p* = 0.0003**
7. Troponin, ng/mL (mean ± SD)	4 +/− 6.3	9.9 +/− 11	*p* = 0.1206
Postoperative 7th day):			
1. WBC, ×10^9^/L (mean ± SD)	9.1 +/− 4.9	9.2 +/− 2.6	*p* = 0.4047
2. Neutrophils, ×10^9^/L (mean ± SD)	5.1 +/− 1.9	5.5 +/− 2.1	*p* = 0.3956
3. Lymphocyte, ×10^9^/L (mean ± SD)	2.5 +/− 3.4	2.4 +/− 1.7	*p* = 0.4216
4. Hb, mmol/L (mean ± SD)	6.9 +/− 0.6	7.1 +/− 0.7	*p* = 0.1483
5. Plt, ×10^9^/L (mean ± SD)	305.7 +/− 93.4	283.5 +/− 70.6	*p* = 0.2592
6. NLR (mean ± SD)	2.7 +/− 1.4	3 +/− 1.9	*p* = 0.6694
7. Troponin, ng/mL (mean ± SD)	0.2 +/− 1.0	0.1 +/− 0.5	*p* = 0.7638

Hb—hemoglobin, Me—mediana, NLR—neutrophil to lymphocyte ratio, Plt—platelets, Q—quartile, WBC—white blood count.

**Table 3:** Univariable and multivariable Cox’s proportional hazard regression analysis.

	Univariate Analysis			Multivariate Analysis		
Parameter	HR	95%CI	*p*-Value	HR	95%CI	*p*-Value
Gender M/F Ref. =F	2.55	0.60–10.79	0.2034			
Age	1.03	0.97–1.07	0.2823			
Concomitant diseases:						
Arterial hypertension	1.32	0.49–3.51	0.5736			
DM	0.98	0.43–2.23	0.9668			
Stroke	14.07	6.34–31.22	**<0.0001**			
Hypercholesterolemia	1.35	0.59–3.11	0.4752			
PAD	3.9	1.77–8.60	**0.0007**			
Surgical indication:						
LM disease	0.85	0.39–1.85	0.6906			
3 vessels disease	1.14	0.52–2.50	0.7314			
2 vessels disease	1.74	0.80–3.81	0.1597			
Echocardiographic results:						
LV diameter (mm)	1.08	1.02–1.16	**0.0191**			
LVEF (%)	0.88	0.85–0.91	**<0.0001**	0.92	0.87–0.95	**<0.0001**
Preoperative:						
(mean ± SD)						
WBC, ×10^9^/L	0.92	0.77–1.09	0.3248			
Neutrophils, ×10^9^/L	0.94	0.75–1.19	0.6364			
Lymphocyte, ×10^9^/L	0.78	0.45–1.35	0.3734			
Hb, mmol/L	0.98	0.62–1.52	0.9218			
Plt, ×10^9^/L	1	0.99 -1.01	0.7882			
NLR	1.04	0.84–1.29	0.6986			
Troponin, ng/mL	0.91	0.34–2.41	0.8498			
Postoperative 1st day:						
(mean ± SD)						
WBC, ×10^9^/L	1.05	1.01–1.08	0.0059	1.18	1.07–1.30	**0.0006**
Neutrophils, ×10^9^/L	1.59	1.33–1.89	**<0.0001**	0.36	0.22–0.58	**<0.0001**
Lymphocyte, ×10^9^/L	0.64	0.37–1.10	0.1047			
Hb, mmol/L	1.14	0.61–2.15	0.6848			
Plt, ×10^9^/L	1.01	1.01–1.01	**0.0065**	1.01	1.01–1.01	**0.0038**
NLR	1.47	1.30–1.65	**<0.0001**	1.61	1.18–2.18	**0.0022**
Troponin, ng/mL	0.99	0.99–1.01	0.8088			
Postoperative 7th day:						
(mean ± SD)	1.01	0.93–1.08	0.9178			
WBC, ×10^9^/L	1.11	0.93–1.32	0.2687			
Neutrophils, ×10^9^/L	0.99	0.87–1.12	0.8528			
Lymphocyte, ×10^9^/L	1.53	0.85–2.74	0.1524			
Hb, mmol/L	0.98	0.99–1.01	0.3514			
Plt, ×10^9^/L	1.1	0.88–1.38	0.412			
NLR	0.94	0.56–1.57	0.8105			
Troponin, ng/mL						

Abbreviations: F—female, DM—diabetes mellitus, Hb—hemoglobin, LV—left ventricle, LVEF—left ventricle ejection fraction, M—male NLR—neutrophil to lymphocyte ratio, PAD—peripheral artery disease, Plt—platelets.

**Table 4:** Multivariable Cox’s proportional hazard regression analysis—binary covariates.

Parameter	HR	95%CI	*p*-Value
LVEF ≤ 50	12.56	3.69–42.72	**0.0001**
WBC > 7.6	1.04	0.32–3.45	0.9409
Plt > 350	2.70	1.19–6.15	**0.0180**
NLR > 4.6	9.30	3.60–24.02	**<0.0001**

Abbreviations: LVEF—left ventricle ejection fraction, NLR—neutrophil to lymphocyte ratio, PLT—platelets, WBC—white blood count.

## Data Availability

All data will be available under the correspondence e-mail address for 3 years following the publication after justifiable request.
